# Acute effects from the half-squat performed using a repetition versus differential approach in youth soccer players

**DOI:** 10.1186/s13102-022-00413-5

**Published:** 2022-02-09

**Authors:** Diogo Coutinho, Eduardo Abade, Bruno Gonçalves, Sara Santos, Wolfgang Schöllhorn, Jaime Sampaio

**Affiliations:** 1 Department of Sports Sciences, Exercise and Health, University of Trás-os-Montes and Alto Douro, Vila Real, Portugal; 2 Research Center in Sports Sciences, Health Sciences and Human Development, CIDESD, CreativeLab Research Community, Quinta de Prados, Ap. 202, 5000-911 Vila Real, Portugal; 3 University of Maia, ISMAI, Maia, Portugal; 4 Departamento de Desporto e Saúde, Escola de Saúde e Desenvolvimento Humano, Universidade de Évora, Évora, Portugal; 5 Comprehensive Health Research Centre (CHRC), Universidade de Évora, Évora, Portugal; 6 Portugal Football School, Portuguese Football Federation, Oeiras, Portugal; 7 Institute for Training and Movement Science, Johannes Gutenberg-University, Mainz, Germany

**Keywords:** Eccentric training, Jumping performance, Sprint, Change-of-direction ability, Flywheel

## Abstract

**Background:**

Over the last years there have been a wide body of research exploring the best strategies to promote acute enhancements in players’ performance. Despite that, most studies have been focused on adult and elite players, and different results may be identified when considering players from lower levels of performance and belonging to youth categories. In addition, most studies conducted in this domain focused in repetitive movement patterns, and while adding variability has been considered as a useful approach to enhance players’ performance at short and long-term perspectives, less is known regarding it applicability to acute enhance players physical performance. Therefore, this study aimed to compare the acute enhancement effects of performing the half-squat in a flywheel ergometer between a more-repetitive approach (low noise) and a more variable approach (differential learning, high noise) in youth soccer players.

**Methods:**

A total of sixteen players (age = 16.2 ± 0.6 years) was exposed to four conditions in a randomized order: (1) repetitive intervention for 30 s; (2) repetitive intervention for 10-min; (3) differential learning intervention for 30 s; (4) differential learning intervention for 10-min. Each condition consisted in 3 sets of 6 repetitions of eccentric half squats performed in a flywheel ergometer. Countermovement jump, 10 m and 30 m linear sprint, and change-of-direction ability were measured every session at baseline (pre-test) and after each protocol (post-test).

**Results:**

No potentiation effect was observed overall with any of the interventions. In addition, no differences between protocols were found for sprinting. However, the repetitive intervention impaired jumping performance for both 30 s (small effects, *p* ≤ .05) and 10-min intervals (small effects, *p* ≤ .05), as well as in the change-of-direction task for 30 s (*p* ≤ .05).

**Conclusions:**

These results may be due to the players’ low experience in eccentric flywheel training. Despite these findings, individual potentiation responses emerged from both protocols when considering the individual responses, reinforcing the need to establish more personalized approaches.

## Background

Enhancing physical performance is a major goal of any sports training process [1, 2]. With increasing professionalization in soccer, in addition to the historically grown interest in continuous performance potentiation, the enhancement of acute mental and physical performance is also receiving more and more attention. Coaches and sports scientists have been trying to develop strategies to promote acute neuromuscular enhancements [2, 3]. Versatile protocols have been tested to understand how players’ performance may be acutely enhanced, such as adopting maximal isometric actions or dynamic heavy resistance loads [4]. Most protocols have been found to improve lower-limb power and sport specific-performances, such as jumping height [4]. Considering these sport-related improvements, some studies have explored how players’ soccer performance may be enhanced by adopting different re-warmup protocols as potentiation strategies. For example, Abade et al. [5] explored how different re-warm up protocols affected the physical performance of youth elite soccer players. The authors found improvements in jumping and sprinting performance following plyometrics and repeated change-of-direction (RCOD) protocol strategies, while eccentric exercise protocols (Nordic hamstring) decreased jumping performance [5] compared to a control situation where the players have passively rest for 12-min. More recently, a study compared a controlled warm-up with two intervention protocols based on squat movements under ~ 60% and ~ 90% load of 1 repetition maximum (1RM), respectively, while also accounting with different levels of soccer experiences (i.e., national and regional level) [6]. In sum, the repeated sprint revealed higher improvements when using the heavier load and mostly for the national level players [6]. Overall, these studies can help coaches to decide about he type of warm-up protocol to enhance players’ performance and also to indicate that protocols are highly sensitive to the personal history of the players and in consequence induce distinct effects. In this regard, most of the studies that are trying to understand enhanced acute performances have been focused on elite soccer players, therefore, much less is known regarding its effects in players from lower competitive levels.

It has been highlighted that the acute potentiation protocols effects may be amplified when using eccentric protocols in iso-inertial devices (i.e., flywheel ergometers) [3, 7–9]. Improvements in counter-movement jumping (CMJ) performance [7, 8], sprinting performance [9], and the ability to change-of-direction [8] have been found while using these devices as potentiation protocols. The advantage of using these inertial devices seems to be related to the mechanical load performed during the eccentric phase. Nevertheless, research exploring the performance potentiation effects using these inertial devices are still scarce [3], especially when considering youth soccer players.

Overall, most of the studies adopting potentiation protocols have been applied under more repetitive and traditional movement exercises, whereas the performers attempt to execute every repetition identically without voluntary variations. Recently, the differential learning (DL) approach, has been suggested as a potential method for strength training [10]. The practical consequences of the DL approach have been derived from system dynamic principles where fluctuations are attributed a central role in the change of states [11, 12]. By amplifying the fluctuations at each movement, self-organizing adaptive mechanisms are triggered in the perception–action system [11–13]. More specifically, this approach adds continuous perturbations that challenge the player to search for more effective states [14, 15]. Improvements in physical performance sustained by DL approaches have been addressed [16]. For instance, players have shown to improve their jumping and sprinting performance, as well as, the ability to perform change-of-directions after being exposed to a 2-month intervention underpinned on DL. Similar findings were identified by a recent study that exposed youth basketball players to differential resistance training over 7-weeks and found improvements in change-of-direction ability compared to the repetitive-based approach [17]. From the acute perspective, the DL approach has also shown better results in jumping performance compared to more repetitive-based interventions [18]. Despite the improvements found with DL interventions on the players’ physical performance, effects of acute enhancements are still an open question. Thus, the study aimed to compare the acute performance enhancement between a repetitive-based and DL based-approach on youth amateur soccer players CMJ performance, sprinting time and RCOD ability and, second, to explore how different time-periods (30 s and 10-min) modify these effects. Based on the players’ amateur profile, it was hypothesized that potentiation effects would be identified only for the jumping performance. In addition, it was expected that the DL intervention would contribute to better performance compared to the repetitive-based approach.

## Methods

### Subjects

Sixteen youth male soccer players from the same Portuguese club participated in this study (age = 16.2 ± 0.6 years; Height = 173.1 ± 8.4 cm; Weight = 65.3 ± 6.6 kg; Years of experience = 8.3 ± 2.8 years). All players were familiar to weightlifting training at the club once per week, and therefore, all the participants had at least 4-month experience in this type of training and had no injury during the last 3 months previous to the study. Informed consent was provided to the coaches, players, and their parents and the club before the beginning of the study. Both the players and their parents gave their written informed consent, and they were fully informed of their rights to withdraw from the study at any time without providing any reason. The study protocol followed the guidelines and was approved by the local Ethics Committee and conformed to the recommendations of the Declaration of Helsinki.

### Design

**Fig. 1:**
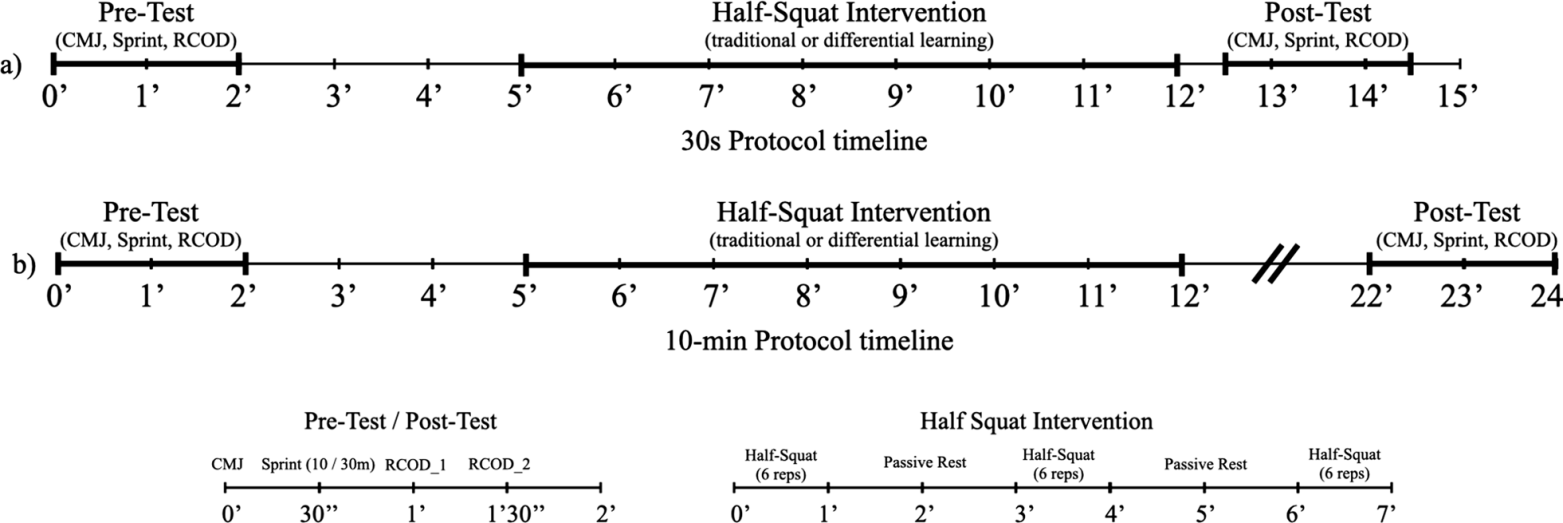
Schematic representation of experimental design. The protocols were performed in a randomized order across 4 testing sessions, whereas **a** corresponds to the time-period of 30 s, while **b** consists in the time-period of 10-min

A crossover-controlled design was performed during the middle of the competitive season. Accordingly, the protocols were performed over 4 testing sessions, one per week, and using a randomized sequence, i.e., all players performed the same randomized protocol in each day. This procedure was adopted because of the participants lesser experience with eccentric strength, while also, to maintain similar routines as those found in association football where players are often exposed to one session per week of increased strength training load [19]. The protocols had similar durations, however the type of protocol was modified as the following (see Fig. 1): (1) repetitive intervention; (2) DL intervention. In addition, both protocols were tested according to 2 time-periods: (1) 30 s after performing the last set; (2) 10-min after performing the last set. Two weeks before the first testing session, players performed 3 familiarization sessions separated by at least 72 h, where they were fully instructed regarding the protocols of the study and were exposed to the repetitive and DL intervention. The number of familiarization sessions were selected upon the time where players’ technical execution on the flywheel becomes stable. During the testing days, players were asked to not engage in other physical activities.

### Methodology

All testing sessions were performed at the same time of the day (18:00 PM), which consisted in their usual training routines. Before each testing session, the players performed a 10-min warm-up, consisting of 5-min of slow jogging, followed by dynamic mobility exercises focused on movements associated with the squat and countermovement jump. Following the warm-up, players performed the pre-test evaluations (one for each testing day, n = 4), and after a few recovering periods of 3-min, the protocol corresponding to each day was performed. During the 4 testing sessions, players performed 3 sets of 6 maximal bilateral coupled concentric and eccentric muscle actions [7] in the YoYo half-squat exercise based on a flywheel device (RSP Squat®, Pontevedra, Spain) with a recovery of 120-s between sets [9]. The number of masses installed allowed to adjust the overall inertial and we used 4 masses, contributing to an inertia of 524.55 kg/cm^2^. After the protocol, the players were re-tested according to the following time-periods: 30 s after the finishing the protocol; or 10-min after finishing the protocol.

### Intervention protocols

In the repetitive approach, the players performed the 3 sets of 6 repetitions under regular half-squat movements. In contrast, during the DL intervention, every repetition from each set consisted of a different type of half-squat movement that can be seen in Table 1. In both protocols, players were instructed to perform squat exercise at maximum concentric velocity. The DL movement variations were chosen in order to slightly increase the biomechanically measurable fluctuations of leg and trunk muscle activations that are constantly accompanied by neuro-muscular challenges related to posture and balance control. Exemplarily explained: when lifting the arms in front during a normal squat with upright body posture, the centre of gravity of the arm and hand segments is shifted forward, then other body parts will move backwards in order to keep the centre of gravity above the area of support. By shifting most probably the lower trunk backwards the activation of the gluteal and thigh muscles, and with this the calf muscle as well, will experience higher fluctuations in activity than during normal squats [20]. These fluctuations of corresponding muscle activation are intentionally larger than the normal variations that can be measured during repetitive squats. Thereby the relative load distribution on the leg muscles can be varied despite the same external load. In sum, the idea of differential strength training is the combined holistic challenge of the muscular and neuronal control systems by means of increased fluctuations.

**Table 1:** Representation of the DL movements during the Half-Squat at the RSP Squat device

Repetition	DL movements (1st set)	Repetition	DL movements (2nd set)	Repetition	DL movements (3rd set)
1	Right arm in extension	7	Right arm overhead	13	Right arm in the chest
2	Left arm in extension	8	Left arm overhead	14	Left arm in the chest
3	Both arms in extension	9	Both arms overhead	15	Both arms in the chest
4	Right arm abduction	10	Both arms down	16	Right hand tennis ball air throw
5	Left arm abduction	11	Receive tennis ball right hand	17	Left hand tennis ball air throw
6	Both arms in abduction	12	Receive tennis ball left hand	18	Both hands tennis ball air throw

## Data collection

### Jump test (countermovement jump)

The counter-movement jump (CMJ) was assessed to inspect the neuromuscular impact of the repetitive and DL protocols through the jumping height (cm) and was measured before and after the intervention according to the time-period (30 s and 10-min, respectively). During the CMJ, players were instructed to jump with the hands on the hips to avoid the arm-swing effects [21]. The CMJ was assessed using a portable optical timing system (Optojump, Microgate, Bolzano, Italy), which has been shown to provide low standard errors of measurement (0.8 cm) [22].

### Sprint tests (10 and 30 m sprint running)

Sprint performance was assessed before and after the intervention with one 30 m maximum sprint. The time was measured using three pairs of photoelectric cells (Optojump, Microgate, Bolzano, Italy), positioned at 0 m, 10 m and 30 m and at a height of 1 m, allowing to record both sprints in just one trial. The participants started the sprint from an upright standing position with the front foot placed at -0.5 m before the first timing gate [5]. Sprinting performance was captured 30 s after the CMJ assessment. Measurement errors for single light barriers have been found to be of 0.035 s for 10 m sprint and 0.029 s for 30 m sprint [23].

### Repeated change-of-direction test

The repeated change-of-direction test (RCOD, [24]) was measured before and after the intervention according to the respective time-periods by performing 2 sprints. The sprint time was recorded using the photoelectric cells (Optojump, Microgate, Bolzano, Italy) positioned in the beginning and ending lines (0 and 20 m), in 1 m height. Players were instructed to start the sprint from an upright standing position and with the front foot at − 0.5 m behind the first pair of cells.

### Statistical analysis

Individual and mean changes from pre- to post-test for all considered protocol activities were graphically represented and the variation from each moment was expressed in percentage variation (mean ± SD). In addition, both intra-day (between-players) and day-to-day variability (within-players) performance was assessed as typical error and expressed as a coefficient of variation, CV % [25].

All data were assessed for outliers and assumptions of normality and were processed to fit analysis of covariance (ANCOVA), with post-test values as the dependent variable and pre-test values as covariate. This procedure was selected as the players pre-test performance was measure on each testing day (n = 4), rather than having an unique day that would act as baseline. For each ANCOVA, partial eta-squared (η^2^) was calculated. Values of 0.01, 0.06 and above 0.14 were considered as small, medium and large, respectively [26]. Statistical significance was set at *p* < 0.05 and calculations were carried out using SPSS software V24.0 (IBM SPSS Statistics for Windows, Armonk, NY: IBM Corp.).

Complementary, to realize the magnitude effects between protocols interventions on players’ performance measures, the data were analyzed using a specific spreadsheet for pre-post crossover trial. Accordingly, these effects were estimated in percent units based on log-transformation and uncertainty in the estimate was expressed as 95% confidence limits. Also, standardized (Cohen) mean differences and respective 95% confidence intervals were also computed as the magnitude of observed effects, and thresholds were 0.2, trivial; 0.6, small; 1.2, moderate; 2.0, large; and 0.2.0, very large [25].

## Results

**Fig. 2:**
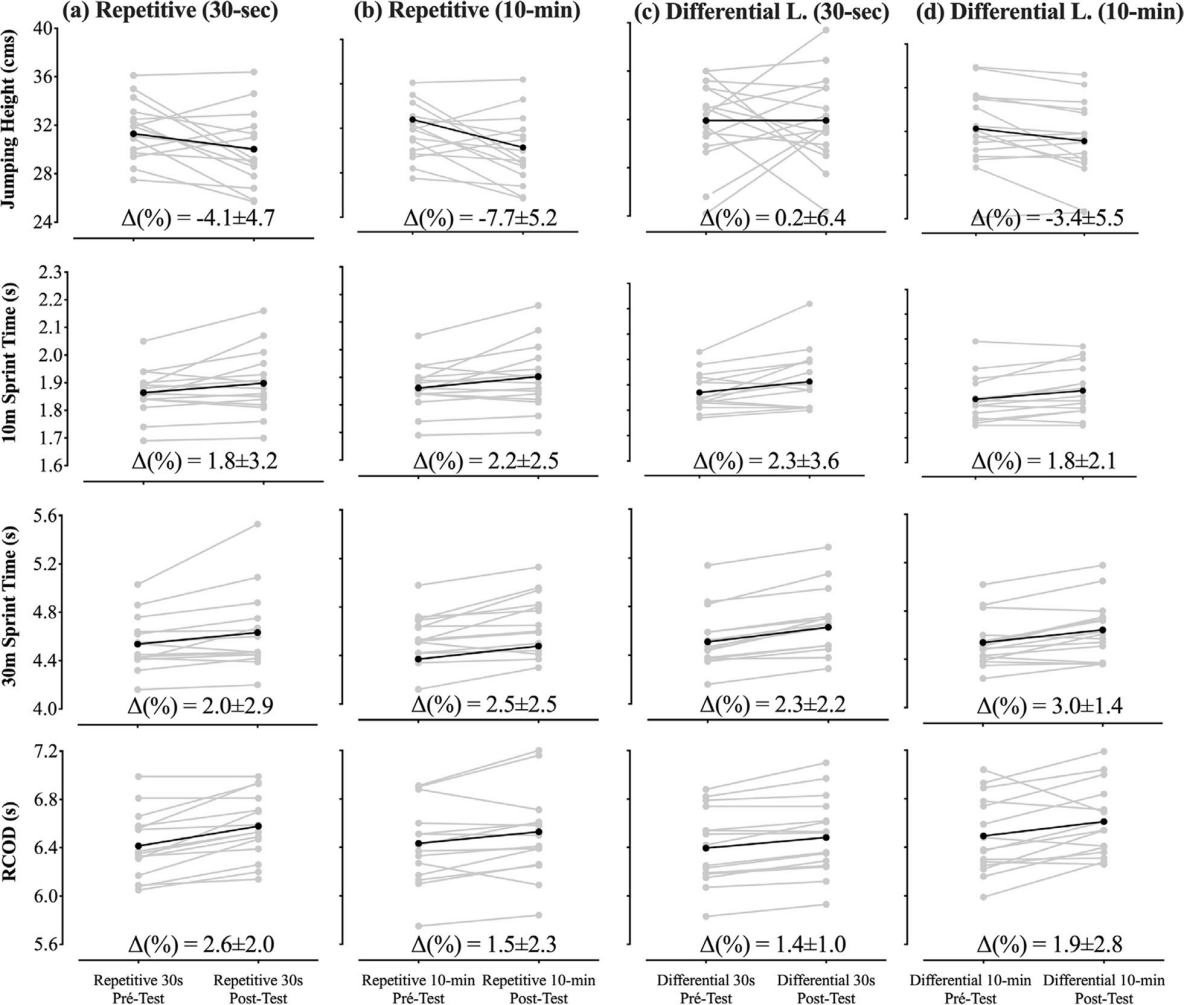
Acute effects after the repetitive and DL intervention of 30s (3a,3c) and 10-min time-period (3b,3d). Percentage variations (Δ%) are expressed as mean ± std for intra-day variability. Note: grey solid lines indicate responses of individual participants; black lines indicate mean value. *RCOD* Repeated change-of-direction; **a** and **b** represents the individual and mean change from pre to post test in physical performance during the repetitive intervention during the 30-sec (**a**) and 10-min (**b**), while the (**c**) and (**d**) represents the individual and mean change from pre to post-test during the differential learning intervention in the 30-sec (**c**) and 10-min (**d**) periods

Individual and mean changes from pre to post interventions according to the physical performance tests are shown in Fig. 2. The intra-day (between-players) pre-post performance revealed ~ 10% variation in the jumping performance (CV %, mean ± SD, 10.3 ± 0.7), ~ 7% in 10 m sprint (7.2 ± 0.9), ~ 8% in 30 m sprint (8.3 ± 3.4) and ~ 5% variation in the RCOD (4.8 ± 0.5). Regarding inter-day variability (within-player), pre to post performances showed ~ 6% variation in jumping performance (5.7 ± 1.7), ~ 3% variation in 10 m sprinting (2.6 ± 1.0), ~ 4% in 30 m sprinting (4.0 ± 7.4) and lastly ~ 3% variation in the RCOD performance (3.2 ± 1.0).

**Fig. 3:**
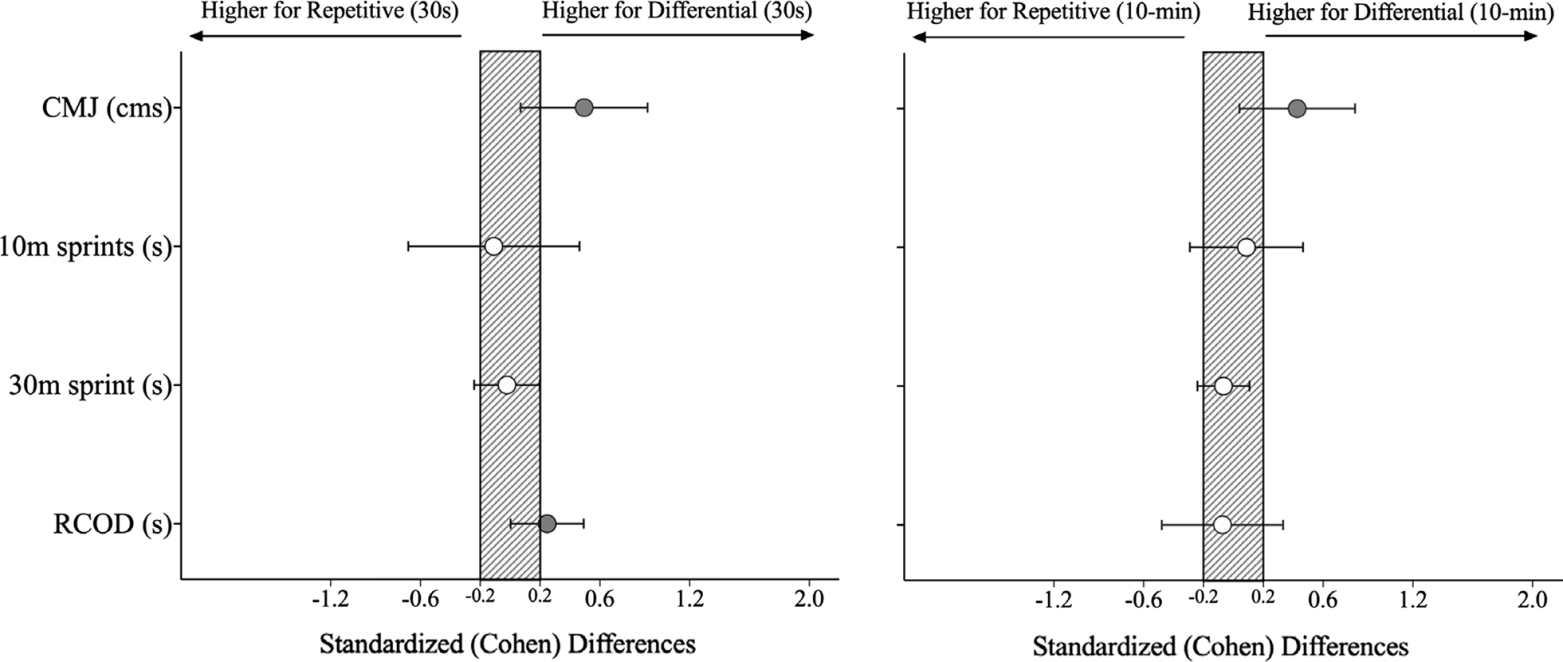
Standardised (Cohen) differences between the repetitive and differential learning intervention for the physical performance variables according to the time interval (30 s and 10-min). Error bars indicate uncertainty in the true mean changes with 90% confidence intervals

The differences between the training interventions according to the time interval (30 s and 10-min) can be found in Tables 2, 3, and Fig. 3. Significant differences were only identified for the jumping performance and repeated change-of-direction task. Accordingly, while lower jumping performances were found after both protocols, the repetitive intervention promoted higher decrements compared to the differential intervention in both 30 s (small effects; *F* = 4.46, *p* ≤ 0.05; η^2^ = 0.137) and 10-min (small effects; *F* = 4.71, *p* ≤ 0.05; η^2^ = 0.140). In addition, players increased the time required to perform the change-of-direction task during 30 s time (*F* = 4.46, *p* ≤ 0.05; η^2^ = 0.014) following the repetitive intervention compared to the differential learning protocol.

**Table 2:** Descriptive (mean ± SD) and inferential analysis for the considered variables between the repetitive and DL approaches according to the 30 s time-period

Variables	Repetitive approach		Differential learning		Difference in means (raw; ± 90% CL)	*F*	*P*	*η*^2^
	Pre-test (30 s)	Post-test (30 s)	Pre-test (30 s)	Post-test (30 s)				
	(Mean ± SD)	(Mean ± SD)	(Mean ± SD)	(Mean ± SD)	Repetitive vs intervention			
Countermovement jump (cms)	31.29 ± 2.67	30.03 ± 3.14	31.9 ± 3.50	31.58 ± 3.57	1.58 ± 1.23	4.46	**.044**	0.137
10 m sprint (s)	1.86 ± 0.08	1.90 ± 0.12	1.87 ± 0.07	1.91 ± 0.12	0.01 ± 0.05	0.11	.743	0.004
30 m sprint (s)	4.54 ± 0.22	4.63 ± 0.33	4.54 ± 0.20	4.64 ± 0.24	0.01 ± 0.09	0.043	.837	0.002
Repeated change-of-direction task (s)	6.41 ± 0.27	6.58 ± 0.27	6.40 ± 0.31	6.48 ± 0.32	− 0.08 ± 0.08	4.68	**.039**	0.143

Values in bold represent significant differences at *p* < .05

**Table 3:** Descriptive (mean ± SD) and inferential analysis for the considered variables between the repetitive and DL approaches according to the 10-min time-period

Variables	Repetitive approach		Differential learning		Difference in means (raw; ± 90% CL)	*F*	*P*	*η*^2^
	Pre-test (30 s)	Post-test (30 s)	Pre-test (30 s)	Post-test (30 s)				
	(Mean ± SD)	(Mean ± SD)	(Mean ± SD)	(Mean ± SD)	Repetitive vs intervention			
Countermovement jump (cms)	32.79 ± 2.72	30.28 ± 3.14	32.26 ± 3.95	31.12 ± 3.59	1.36 ± 1.23	4.41	**.038**	0.140
10 m sprint (s)	1.86 ± 0.08	1.9 ± 0.09	1.86 ± .09	1.89 ± 0.1	− 0.01 ± 0.03	0.222	.641	0.008
30 m sprint (s)	4.63 ± .33	4.66 ± 0.25	4.52 ± 0.25	4.66 ± 0.27	0.03 ± 0.07	0.618	.439	0.022
Repeated change-of-direction task (s)	6.43 ± 0.33	6.53 ± 0.37	6.49 ± 0.32	6.61 ± 0.3	0.02 ± 0.13	0.211	.649	0.008

Values in bold represent significant differences at *p* < .05

## Discussion

The present study aimed to explore the youth players acute responses to two methods that potentially enhance performance. Therefore, we compared the short-term effects of repetitive repetition oriented and differential learning interventions during the half-squat exercise on a flywheel device on youth soccer players’ jumping, sprinting and change-of-direction performances. We also inspected the effects of these interventions according to two time-periods: 30 s and 10-min. It was expected that the players improved some of the physical tests, mainly the jumping performance because of the similarity of movement with the squat performed on the flywheel. In contrast to our expectations, players’ jumping and change-of-direction performances revealed decrements following both protocols, which may be related to players’ little experience in eccentric flywheel training. In addition, it was hypothesized that players would how the worst performances following the repetitive-based approach compared to the differential learning intervention. Accordingly, the repetitive training preparation induced higher decreases in performance, mainly in jumping at both 30 s and 10-min and in the RCOD at 30 s.

Literature has already indicated that performing flywheels squats may acutely improve jumping capacity in university-level soccer players (~ 20 years of age) when performing more than 1 set and when accounting for an interval between the exercise and tests of 6-min, while 3-min time interval may have trivial effects [7]. Such results are in accordance with 30 s period jumping performance for the repetitive intervention in the present study. However, while most studies have found improvements in jumping performance following a time interval of at least 3 to 7 min [27], in this study players’ acute performance were still lower in the post-intervention after the 10-min time-interval compared to the pre-test. Accordingly, performance enhancement following eccentric protocols seems to be dependent on the balance between fatigue and potentiation. From this perspective, lower level athletes (i.e., less experienced in weight training) appear to be more susceptible to fatigue in contrast to more trained athletes [27]. In consequence, because recovery periods are determinant for performance outcome, longer time intervals (i.e., above than 15-min) may be required to promote acute enhancements in recreationally players [28]. Nevertheless, it is important to note that players’ responses are highly individual (see Fig. 2) [29], and despite revealing less effects in short-term, there seems to be potential to improve their performance potentiation following more long-term interventions [30].

Despite both protocols presented decrements following the half-squat intervention, smaller decrements were identified following the differential learning intervention. These results may be possibly related to more neuromuscular and coordinative variations despite same external load during the DL intervention since the players were required to perform more complex and different movements at every single repetition. A previous exploratory study compared the effects of a repetitive and a DL strength intervention by performing two sessions per week in jumping performance [10]. While the repetitive intervention trained with an additional weight of 60 to 97.5% of their Single Repetition Maximum (1RM), the DL group trained with only 60% of their 1RM. After the training intervention, the results showed similar improvements in both groups, suggesting that positive effects can be achieved with lower external loads by taking advantage of the internal levers, muscle fibre alignments and accompanying neuronal control [10]. Other studies have also found superior performance from DL interventions over repetitive approaches in jumping performance both from short-term [18] and long-term perspectives [16]. Apparently, these enhancements may be related to a more variable activity in the muscles, as well as to exposing the players to a more variable time under tension compared to more repetitive approaches [31]. In addition, the players were required to perform each repetition under a different movement pattern during the DL, that may be possibly allowed to distribute the load over more muscle groups, and decreasing the likelihood of inducing fatigue in the same muscle groups as during the repetitive intervention [20]. In contrast, it is possible that the same force output was achieved by players during the repetitive intervention with less variation that is accompanied by faster fatigue. The more similar the movements the higher the probability of activating the same muscle fibres, and so the higher the probability of fatigue due to overloading. In fact, less experienced players seem to be more affected by eccentric based protocols [5, 27], whereas the fatigue seems to overcome potentiation, which may have contributed to lower jumping performance after repetitive approach.

Previous research exploring the potentiation effects using eccentric protocols found improvements in change-of-direction performances in youth elite soccer players [9]. Similarly, a more recent study using physically active male participants found improvements in the RCOD performance with moderate and high loads based on eccentric protocols [8]. In addition, the results revealed better performances after 6-min rest compared to 3-min and 30 s, respectively [8]. In this study, both protocols resulted in worst performances following intervention. The type of participants (e.g., youth, vs professional, vs physically active adults) and the type of RCOD test [32] may justify such results. In our study higher decrements were found following the repetitive intervention compared to the differential learning protocol during the 30 s period. The ability to successfully perform the RCOD seems to be dependent of the level of fatigue, and as previously stated, performing the half-squat under more repetitive movements may have amplified the fatigue compared to the DL intervention which may distribute the load toward more muscle groups, and consequently, revealing less decrements than the repetitive approach.

Overall, players’ performance was maintained or impaired following the eccentric protocols. However, when considering the Fig. 2 it can be depicted that some players were able to improve their jumping, sprinting or change-of-direction ability with the interventions applied. These findings are consistent with those available in the literature that also highlighted the importance of considering different and singular responses. Therefore, rather than exploring general guidelines for potentiation strategies, sports scientists and coaches may implement individual strategies adjusted to each player profile. In fact, following the implementation of a 20-weeks endurance training intervention, Bouchard et al. [33] found an extraordinary variability in participants’ responses to the intervention. Based on this, it seems crucial that coaches and sports scientists develop individual strategies while considering the type of protocol, the type of task and the recovery time, mainly when dealing with athletes with lower experience. In such circumstances, less experienced players seem to be less susceptible to potentiation, especially when exposed to eccentric loads. When considering the long-term perspective, coaches must test players responses to different protocols and time-periods during a transitory period (i.e., without competition) to allow a better planning of the short and mid-term stimulus without compromise the players’ behavior during competitive performances. In addition, during an early eccentric stimulus, the differential learning approach may be more suitable as it allows to introduce the players to eccentric load with less decrements compared to a more repetitive-based approach, mainly under shorter timescales (e.g., 30 s).

The present study provided findings regarding the effects of the performance potentiation according to different time-periods and training interventions. Despite the insights resulting from this study, some limitations must be acknowledged. Firstly, players’ age and sports experiences may refrain from achieving stronger inferences. In fact, players from different levels (i.e., professionals vs amateurs; experienced in weightlifting vs non-experienced) may adapt differently under these protocols. In addition to this, the external load was kept constant to all players (524.55 kg/cm^2^) which may be excessive for the present participants, amplifying the fatigue and preventing the onset of potentiation. Also, considering the present sample, adopting more intermediate measures and additional rest-time periods would allow to better understand the activation effects. Considering these limitations, future studies may explore how players from different playing levels (professional vs amateurs), age groups (U17, U19, U23) and gender (male vs females) are affected, particularly by differential eccentric performance enhancement protocols, while accounting for individual adjusted load.

## Conclusions

Overall, both protocols contributed to acute performance decrements.. While improvements in players’ performance were expected, the decrements may be linked with the type of exercise that was more oriented towards short-term muscle metabolism, the low performance level, and little experience of the players from this study. That is, when less experienced players perform eccentric protocols, fatigue may overcome the potentiation effects. The results found by the repetitive intervention support this finding, as higher decrements in jumping performance at 30 s and 10-min and change-of-direction at 30 s were found following this protocol, as in accordance with our expectations. Accordingly, the increased fluctuations performed under the DL protocol may have led the players to complete the pre-load with lower intensities and distributing the load over more muscle groups, attenuating the decrements. Despite that, individual potentiation responses emerged from both protocols and time periods, which reinforces the need to establish individual and adequate prescriptions guidelines.

## Data Availability

The datasets used and/or analysed during the current study are publicly available from the corresponding author on reasonable request.

## Abbreviations

RCOD: Repeated change-of-direction
1RM: 1 Repetition maximum
CMJ: Counter-movement jump
DL: Differential learning
